# Recent Pre-Clinical Advancements in Nuclear Medicine: Pioneering the Path to a Limitless Future

**DOI:** 10.3390/cancers15194839

**Published:** 2023-10-03

**Authors:** William Echavidre, Daniel Fagret, Marc Faraggi, Vincent Picco, Christopher Montemagno

**Affiliations:** 1 Biomedical Department, Centre Scientifique de Monaco, 98000 Monaco, Monaco; wechavidre@centrescientifique.mc (W.E.); vpicco@centrescientifique.mc (V.P.); 2 Laboratory of Bioclinical Radiopharmaceutics, Universite Grenoble Alpes, CHU Grenoble Alpes, Inserm, 38000 Grenoble, France; daniel.fagret@univ-grenoble-alpes.fr; 3 Nuclear Medicine Department, Centre Hospitalier Princesse Grace, 98000 Monaco, Monaco; marc.faraggi@chpg.mc

**Keywords:** nuclear medicine, theranostics, oncology, immunotherapies

## Abstract

**Simple Summary:**

This review summarizes recent advances in the nuclear medicine theranostic approach. It covers the repurposing of historical radiotracers for new indications and highlights new radiotracers in solid malignancies. Additionally, the potential of combining theranostics with immunotherapy is explored, underscoring the promise of personalized and targeted therapies. The progress in theranostics signifies an exciting era in nuclear medicine with potential benefits for diverse cancer types.

**Abstract:**

The theranostic approach in oncology holds significant importance in personalized medicine and stands as an exciting field of molecular medicine. Significant achievements have been made in this field in recent decades, particularly in treating neuroendocrine tumors using 177-Lu-radiolabeled somatostatin analogs and, more recently, in addressing prostate cancer through prostate-specific-membrane-antigen targeted radionuclide therapy. The promising clinical results obtained in these indications paved the way for the further development of this approach. With the continuous discovery of new molecular players in tumorigenesis, the development of novel radiopharmaceuticals, and the potential combination of theranostics agents with immunotherapy, nuclear medicine is poised for significant advancements. The strategy of theranostics in oncology can be categorized into (1) repurposing nuclear medicine agents for other indications, (2) improving existing radiopharmaceuticals, and (3) developing new theranostics agents for tumor-specific antigens. In this review, we provide an overview of theranostic development and shed light on its potential integration into combined treatment strategies.

## 1. Introduction

In 1907, Paul Ehrlich, a distinguished Nobel Prize laureate, introduced the groundbreaking scientific concept of the “magic bullet”. Magic bullets were envisioned as drugs possessing remarkable biological properties, acting as precision-guided missiles, obliterating disease foci while sparing surrounding healthy tissue [1,2]. Even after a century has passed, this visionary concept remains highly relevant in modern oncology: developing specific, efficient, and nontoxic antitumor agents to enhance patient care [3].

The introduction of cytotoxic chemotherapy, followed by the first targeted therapies in the 1980s, was considered a radical breakthrough in oncology. Both approaches have significantly elevated cancer patients’ survival rate and improved their quality of life [4,5,6]. In recent decades, in tandem with strides in genomics and proteomics, the identification of molecular events driving tumorigenesis led to the subsequent development of new therapeutic agents [7]. One such groundbreaking development is the use of tyrosine kinase inhibitors (TKIs), a class of pharmacological agents that interrupt the transduction signal of protein kinases. The arsenal of TKIs in cancer therapy continues to expand exponentially, with about 50 Food and Drug Administration (FDA)-approved TKIs to date [8].

Concurrently with the remarkable progress of TKI treatments, immunotherapeutic approaches have revolutionized the field of oncology in recent years. Immune checkpoints inhibitors (ICIs), exemplified by antibodies against cytotoxic T-lymphocyte antigen 4 (CTLA-4) and programmed death-1 (PD-1), have emerged as a pivotal turning point in cancer therapy [9,10,11]. Despite these momentous advancements in the therapeutic arena, drug resistance remains a significant impediment to achieving long-term responses in cancer patients [12,13,14]. Consequently, there is an urgent need for alternative therapies to prolong progression-free survival (PFS) and overall survival (OS).

Over two millennia ago, Hippocrates emphasized the importance of selecting the “*the right treatment for the right diagnosis*”, which can be seen as the premise for the era of personalized medicine. In today’s context, nuclear medicine has emerged as a valuable tool in personalized medicine, enabling population selection, prediction of treatment response, and targeted tumor eradication [15,16]. At the heart of this approach lies “theranostics”, a neologism composed of the words *diagnostics* and *therapeutics*, aligning perfectly with the fundamental principles of personalized medicine. The core idea is to select the most suitable molecule (diagnostic and therapeutic) for the right patient, to improve treatment outcomes, and to minimize toxicity. Theranostics relies on whole-body molecular imaging to assess the expression and the accessibility of the target, followed by radioligand therapy, using the same molecule or a similar but differentially radiolabeled one to image and irradiate tumor cells, while sparing healthy surrounding tissue [17]. The potential of this approach has garnered positive evaluations in clinical practice in cases of prostate cancer (PC, ^68^Ga-PSMA-11/^177^Lu-PSMA-617) and neuroendocrine tumors (NET, ^68^Ga-DOTA-TATE/^177^Lu-DOTA-TATE), leading to FDA approval [18,19].

In 2023, we find ourselves at the nascent stages of the golden age of theranostics. Numerous radiotracers that are dedicated to both imaging and radionuclide therapy are currently under pre-clinical and clinical trials for solid and hematologic malignancies [20,21,22,23,24]. For this review, our focus was specifically on recent developments in solid tumors, spanning from 2020 to the present.

This new era of theranostics unfolds through several strategic approaches: (1) a repositioning strategy (exploration of existing drugs in novel applications), (2) a modification of existing molecules either by modifying/optimizing the carrier or by changing the radioisotopes, and (3) the development of new molecules. The latest has recently been implemented with the development of radiotracers dedicated to antitumor immunity imaging and can be considered to be a tool for the stratification ICI patients and monitoring their efficacy.

In the past few years, a growing understanding of the molecular effects of radiotherapy (RT) on the tumor microenvironment has led to the exploration of exciting new possibilities in combination therapies. Indeed, radiation shapes the tumor microenvironment and facilitates immune cells’ infiltration. This phenomenon justifies the rationale of combined RT and ICIs [25]. Therefore, we finally shed light on the powerful antitumor effect generated by this synergistic combination.

## 2. Pre-Existing-Based Molecules 

The recent approvals of Luthatera^®^ for adult patients with advanced neuroendocrine tumors (NETs) and that of Pluvicto^®^ for advanced PCs have laid a strong foundation for the use of peptide receptor radionuclide therapy (PRRT). Phase III clinical trials conducted with both therapies for these specific indications have demonstrated their safety and their efficacy in terms of PFS and OS when compared to standard care [26,27]. These approvals mark significant milestones in the field of PRRT. The first crucial step in this theranostic approach involves the use of a molecule labeled with ^68^Ga (^68^Ga-DOTA-TOC and ^68^Ga-PSMA-11) for the quantitative imaging of tumor antigen expression [28,29]. Positive enrichment of the ^68^Ga-labeled molecule in tumor, detected by positron emission tomography (PET) scan, is followed by the administration of the same or a similar molecule labeled with a therapeutic molecule. In both cases, ^177^Lu is used as a therapeutic radionuclide, delivering a tumoricidal dose of radiation.

Despite the undeniable effectiveness of these compounds in early studies, the long-term efficacy of PRRT is still being awaited and is an area of ongoing research.

### 2.1. Repurposing and Optimization of FDA-Approved Drugs

#### 2.1.1. Somatostatin Receptor 2 (SSTR2) Targeting

Imaging of somatostatin receptor expression is a well-established method for assessing the suitability of PRRT for patients with NET, using Lutathera (^177^Lu-DOTA-TATE) [30]. Most NETs exhibit somatostatin receptors (SSTRs) on their cell surface, making them suitable for the use of radiolabeled somatostatin analogues (SSAs) [31]. Lutathera^®^ stands as the pioneering FDA-approved PPRT, following the NETTER-1 trial, which revealed a remarkable PFS rate at month 20 of 65.2% when compared to 10.8% for octreotide alone [26]. However, resistance to PRRT may occur, necessitating the exploration of therapeutic alternatives. Mechanisms such as the downregulation or alteration of SSTRs, enhanced DNA damage repair mechanisms, or treatment-induced hypoxia could contribute to PRRT resistance [32,33]. The list of recently developed molecules targeting SSTR2 is presented in Table 1.

##### DOTA-TATE-Based Radiotracer

A promising strategy to overcome treatment-induced resistance involves the use of higher-energy radioisotopes for radiolabeling DOTA-TATE, including other β- or α-emitters. One such approach is the radiolabeling of DOTA-TATE with Samarium-153 (^153^Sm, ^153^Sm-DOTA-TATE), a β-emitter that emits electrons with higher energy than ^177^Lu and, therefore, with larger range in the tumors [34]. ^153^Sm also emits γ-particles, making this radioelement also suitable for SPECT imaging. In vitro studies conducted on gastroenteropancreatic neuroendocrine tumor cell lines demonstrated a high uptake and internalization of the radiotracer. Moreover, biodistribution studies in mice with gastroenteropancreatic neuroendocrine tumor xenografts showed rapid clearance and strong tumor uptake of the radiotracer [34]. However, the therapeutic potential of ^153^Sm-DOTA-TATE is yet to be fully evaluated and compared with that of ^177^Lu-DOTA-TATE. 

An alternative approach to enhance the efficacy of SST2R-targeting agents is to use α-emitters, which possess higher linear energy transfer (LET) than β-emitters (50–200 Kev/mm vs. 0.1–1 Kev/mm). This leads to a greater number of DNA double-strand breaks and a more potent induction of apoptosis [35]. One such α-emitting radiopharmaceutical, ^225^Actinium-DOTATATE (^225^Ac-DOTATATE), was investigated in a mouse model of lung cancer expressing SSTR2. A single injection of ^225^Ac-DOTATATE resulted in significant growth retardation compared to the control, with no evidence of toxicity [36]. A clinical trial (NCT05477576) is ongoing to evaluate the safety and efficacy of ^225^Ac-DOTATATE in patients with NETs who were previously treated with ^177^Lu-DOTATATE. This targeted α-therapy directed against SSTR2 has also been studied using ^212^Pb-labeling (^212^Pb-DOTAMTATE). Pre-clinical studies showed tumor targeting with a >20% injected dose/g 24 h after injection and a significant effect on OS in mice treated with a single dose of ^212^Pb-DOTAMTATE [37]. The antitumor effect of this radiotracer was further optimized using a chemo-sensitizing agent. The impact of such a bimodal approach is discussed in Section 4 of our review. ^212^Pb-DOTAMTATE was evaluated in 10 patients with NETs in a recent dose-escalation phase I trial (NCT03466216). Based on RECIST 1.1 criteria, an objective response of 80% was observed in patients who experienced progression after Lutathera^®^ treatment, with manageable toxicity [38]. Such promising results warrant further investigations.

##### SARTATE-Based Radiotracer

In addition to the use of targeted α-therapy, which is an exciting area of clinical research, there are other promising methods currently under development. One notable approach involves the use of alternative SSA sarcophagine octreotate (SARTATE) labeled with copper-64 and copper-67 (^64^Cu/^67^Cu-SARTATE). Unlike ^68^Ga and ^177^Lu, the ^64^Cu/^67^Cu couple offer the unique advantage of being a “true” theranostic agent, where the biodistribution between the imaging and the therapeutic agents is identical. The potential of ^64^Cu-SARTATE was initially demonstrated in a lung cancer model, exhibiting superior tumor uptake and retention compared to ^64^Cu-DOTATATE [39]. ^64^Cu-SARTATE was found to be suitable for PET imaging in a prospective trial involving 10 patients with neuroendocrine tumors [40]. The therapeutic counterpart, ^67^Cu- SARTATE, displayed high efficacy in pre-clinical models of PDAC xenografts [41]. Recent studies have further highlighted the potential of this approach in a metastatic model of neuroblastoma [42]. These pre-clinical results encourage its further clinical development, considering the recent approval of ^64^Cu-DOTATATE as an SSTR imaging option [43]. The couple ^64^Cu/^67^Cu-SARTATE was recently evaluated in five patients with unresectable multifocal meningioma and showed great promise as a theranostic pair for this indication [44].

##### Radiotracers Based on SSTR2 Antagonists

A novel and promising avenue to enhance radiation delivery and antitumor effects involves the use of antagonists rather than agonists. Unlike agonists, which can only bind the SSTR in an active state, antagonists can bind active in an inactive state [45]. Higher tumor uptake and radiation delivery was also observed using ^177^Lu-DOTA-LM3 (antagonist) compared to the ^177^Lu-DOTA-TOC (agonist) in pre-clinical mice models [46]. Notably, the median survival of the ^177^Lu-DOTA-LM3-treated mice group was 48.5 days, compared to 19.5 days in the ^177^Lu-DOTA-TOC mice group. These results, along with successful applications of ^177^Lu-DOTA-LM3 in 51 patients with NETs, are encouraging and support further development of this approach [47]. These results are in phase with the superior efficacy of the antagonist ^177^Lu-satoreotide tetraxetan compared to ^177^Lu-DOTA-TATE. ^177^Lu-satoreotide tetraxetan treatment increased the median of survival of mice bearing SSTR-positive tumors and offered favorable safety compared to ^177^Lu-DOTA-TATE [48]. Another promising antagonist, SSTR2-antagonist LM4 (^177^Lu-AAZTA5-LM4), has shown potential as a therapeutic candidate [49].

**Table 1:** A Summary of most recent molecules targeting somatostatin receptors developed for theranostics application.

Radiotracer	Type of Molecule	Disease Model	Study	Reference
^153^Sm-DOTA-TATE	Agonist	CA20948, pancreatic tumor	Pre-clinical	[34]
^225^Ac-DOTA-TATE	Agonist	H727 and H69 cells, Lung neuroendocrine neoplasms	Pre-clinical	[36]
^212^Pb-DOTAMTATE	Agonist	AR42J, pancreatic tumor Neuro-endocrine tumors	Pre-clinical First in humans	[37] [38]
^64^Cu-SARTATE	Agonist	AR42J, pancreatic tumor Neuro-endocrine tumors	Pre-clinical First in humans	[39] [40]
^67^Cu-SARTATE	Agonist	AR42J, pancreatic tumor (metastasis) IMR32, neuroblastoma Multifocal Meningioma	Pre-clinical Pre-clinical Clinical	[41] [42] [44]
^177^Lu-DOTA-LM3	Antagonist	AR42J, pancreatic tumor Neuro-endocrine tumors	Pre-clinical First in humans	[46] [47]
^161^Tb-DOTA-LM3	Antagonist	AR42J, pancreatic tumor	Pre-clinical	[46]
^177^Lu-AAZTA5-LM4	Antagonist	HEK293-SST_2_R transfected cells	Pre-clinical	[49]
^177^Lu-satoreotide tetraxetan	Antagonist	AR42J, pancreatic tumor	Pre-clinical	[48]

In the realm of therapeutic labeling, ^161^Tb has emerged as an intriguing option due to its similar decay properties to ^177^Lu, while also emitting conversion and Auger electrons [50]. ^161^Tb-DOTA-LM3 demonstrated a survival advantage in mice models of PDAC tumors when compared to ^177^Lu-DOTA-LM3 [46].

#### 2.1.2. Prostate-Membrane-Specific Antigen (PSMA) Targeting

In 2022, a significant milestone was achieved with the FDA approval of ^177^Lu-PSMA-617 (Pluvicto^TM^, Advanced Accelerator Applications USA, Inc. (AAA, a Novartis company; Millburn, NJ, USA)) for the treatment of patients with PSMA-positive metastatic castration-resistant prostate cancer (mCRPC) [51]. The FDA approval was based on the compelling results from the phase III VISION trial, which showed the positive impact of ^177^Lu-PSMA-617 on both PFS and OS compared to the control group [27]. Patient eligibility is determined by PET imaging after the injection of ^68^Ga-PSMA-11 [28]. This approach marks a revolutionary advancement in the therapeutic management of mCRPC [52]. The most recently developed molecules are presented in Table 2.

##### Repurposing of ^68^Ga-PSMA-11/^177^Lu-PSMA-617

In addition to its role in PC, PSMA overexpression has been identified in various other tumor types, such as glioblastoma or hepatocellular carcinoma (HCC), making them potential candidates for ^68^Ga-PSMA-11 imaging and ^177^Lu-PSMA-617 treatment [53]. Pilot studies in patients with HCC demonstrated significant uptake in seven patients with multiple liver lesions. Thirty-six out of thirty-seven lesions showed an increased uptake of ^68^Ga-PSMA-11, while only ten lesions were ^18^F-FDG-avid [54,55]. Pre-clinical studies in mice with HCC have shown that a single injection of ^177^Lu-PSMA-617 can suppress tumor growth and prolong survival, laying the foundation for potential clinical applications in HCC treatment [56,57]. In this way, 40 HCC patients were examined with ^68^Ga-PSMA-11, which showed higher specificity than CT for detecting intra- and extra-hepatic lesions, suggesting a potential as a diagnostic agent [58]. Moreover, PSMA has emerged as a promising target for theranostic approaches in glioblastoma multiforme (GBM). PSMA overexpression has been associated with poor prognosis in GBM patients who underwent multimodal therapy [59,60,61]. Additionally, PSMA expression was found in a significant proportion of untreated GBM patients [62]. A high uptake of ^177^Lu-PSMA-617 was observed in a tumor lesion of a 34-year-old patient with recurrent GBM, thus supporting the treatment of such a tumor [63]. Further research and clinical investigations are warranted to fully explore the potential of PSMA-targeted approaches in these indications.

##### Development of ^225^Ac-PSMA-617

Despite that ^177^Lu-PSMA-617 PRRT has shown significant efficacy in many patients with metastatic castration-resistant prostate cancer (mCRPC), up to 30% of patients either do not respond or develop resistance to this therapy [64,65]. To address this challenge, PRRT with α-emitters using ^225^Ac has emerged as a promising alternative. Biochemical (decrease in PSA levels) and radiological responses were shown in 40 patients receiving ^225^Ac-PSMA-617 (three cycles of 9–10 MBq) [66]. ^225^Ac-PSMA-617 was evaluated in 26 mCRPC patients previously treated with ^177^Lu-PSMA-617. Six of the twenty-six patients with PSA progression under ^177^Lu-PSMA-617 experienced a biochemical response after ^225^Ac-PSMA-617 [67]. Sathekge et al. reported a significant positive association between biochemical response and PFS and OS after ^225^Ac-PSMA-617 treatment [68]. However, despite the promising antitumor effects of ^225^Ac-PSMA-617 against mCRPC, xerostomia emerged as a common side effect, affecting the patients’ quality of life. To mitigate this side effect, tandem therapy with ^177^Lu-PSMA-617 and low-dose ^225^Ac-PSMA-617 has been explored, showing objective responses and minimizing xerostomia in late-stage mCRPC [65]. Such results were also confirmed by Langbein et al. [69]. The antitumor and side effects of ^225^Ac-PSMA-617 were extensively reviewed [70,71]. Future randomized clinical trials are awaited to fully validate the potential of ^225^Ac-PSMA-617 in mCRPC. 

##### Other Agents Currently under Development

The long-term toxicity associated with ^177^Lu-PSMA PRRT underscores the importance to develop agents with high antitumor activity and limited uptake by healthy tissues. A series of agents, known as ^177^Lu-L1, showed lower off-target toxicity, with minimal radiotoxicity on the kidneys [72]. ^225^Ac-L1 and ^213^Bi-L1 were recently studied in mice with PSMA+ tumors and showed uptake in PSMA+ tumors and high tumor-to-kidney ratios. Notably, ^225^Ac-L1 demonstrated increased survival benefits in a micrometastatic model compared to ^177^Lu-L1, with minimal relative toxicity [73]. Additionally, other peptides, such as PSMA1 and PSMA5, have been labeled with ^211^At for targeted alpha therapy (^211^At-PSMA1 and ^211^At-PSMA5) and recently evaluated for their ability to target and treat PSMA+ tumors. A single-dose administration of ^211^At-PSMA1 or ^211^At-PSMA5 (0.4 MBq) suppressed the tumor growth of lymph node carcinoma of the prostate (LNCaP) cells. The short physical half-life of ^211^At (7.2 h) is expected to limit kidney retention and subsequent injury. Nonetheless, clinical trials are still awaited to fully assess the safety and efficacy of these compounds. A new PSMA-targeted compound, ^211^At-3-Lu, with high stability and rapid clearance from off-target tissues was recently evaluated in mice [74]. Tumor-to-salivary-gland and tumor-to-kidney ratios were shown to be superior to 100 at 24 h post-injection, leading to low off-target toxicity, while the compound retained antitumor efficacy. Additionally, another PSMA-targeting radiotracer, known as ^188^Re-PSMA-GCK01, was radiolabeled with Rhenium-188 (^188^Re). ^188^Re is a high-energy β-emitting radioisotope with a short physical half-life (16.9 h), making it an attractive candidate for PRRT [75]. In a recent first-in-human study, ^188^Re-PSMA-GCK01 demonstrated a distribution pattern similar to that of ^177^Lu-PSMA-617 [76]. However, the therapeutic efficacy of this radiopharmaceutical is yet to be evaluated. 

##### Modifying PSMA-Agents as a Valuable Strategy to Enhance Therapy Efficacy

Another hypothesis to explain the failure of PRRT could be the insufficient dose delivery to the tumor. This can be attributed to the low expression of the target, inadequate retention time, or radioresistance of the tumor. Addressing this challenge has become a focal point of research, and increasing the blood circulation of the ligand has emerged as a promising approach. One strategy is based on the introduction of the Evans Blue (EB), a dye molecule that can reversibly bind to circulating albumin and, thus, increase tumor accumulation of the radiotracer [77]. This approach has been shown to increase the tumor dose of ^177^Lu-DOTA-TATE and improve patient response [78,79]. A first-in-human study carried out on nine patients with mCRPC showed higher tumor accumulation of ^177^Lu-EB-PSMA-617 compared to ^177^Lu-PSMA-617 [77]. ^177^Lu-EB-PSMA-617 was reported to be safe in a dose-escalating study [80]. Furthermore, radioligand therapy with ^177^Lu-EB-PSMA-617 was recently evaluated in patients with adenoid cystic carcinoma lesions (bone, liver, lung, and intracranial metastasis), leading to a remarkable response after three cycles of PRRT (1.85 GBq) [81]. A similar approach with the compound ^177^Lu-LNC1003, another PSMA-targeting agent using an EB moiety, was recently developed and evaluated. Biodistribution studies performed 24 h post-injection showed a fourfold higher tumor uptake (PC xenograft) in the ^177^Lu-LNC1003 group compared to the ^177^Lu-PSMA-617 group [82].

Another ligand called PSMA-TO-1 (also called PSMA-71) has also been developed to increase circulation time, tumor uptake, and radiation delivery. Pre-clinical experiments in mice with metastatic PCs have shown promising results with PSMA-TO-1. Mice bearing LNCaP tumors (C4-2 xenografts) treated with ^225^Ac-PSMA-TO-1 exhibited longer survival compared to those treated with ^225^Ac-PSMA-617, suggesting that PSMA-TO-1 may have superior therapeutic efficacy [83]. However, the higher tumor uptake observed with PSMA-TO-1 was accompanied by a higher uptake in kidney and salivary glands. This off-target uptake in healthy tissues may lead to adverse effects and limit the overall efficacy of this approach.

Other radiotracers based on this strategy are under development, such as PSMA-DA1 or PSMA-NAT-DA-1, which show strong antitumor activity when labeled with ^225^Ac in a pre-clinical setting [84,85]. However, as with any new therapeutic approach, clinical studies are essential to validate their safety and efficacy in human patients. Recent advancements in PSMA-targeted radiotracers have included monoclonal antibodies, which offer the advantage of a limited uptake in salivary glands. The use of such a molecule could overcome xerostomia, which limits the use of radiolabeled small molecules [86]. One notable PSMA-targeted radiotracer in this category is the ^227^Th-BAY 2315497. It was recently studied in PDX models of PRPC [87]. Treatment with ^227^Th-BAY 2315497 showed a potent antitumor effect in cell lines and PDXs, including models that were resistant to standard-of-care drugs. These results support the clinical development of the radiotracer for the treatment of mCRPC; a phase I is currently underway (NCT03724747). ^227^Th-BAY 2315497 was also found to synergically act with Darolutamide, an androgen-receptor inhibitor in PC models [88]. This combination therapy approach has shown recent success and highlights the potential for integrating PSMA-targeted radiotracers with other “cold” therapies for enhanced therapeutic outcomes. Recent successes in combining PRRT and “cold” therapies are discussed in Section 4.

##### Development of ^18^F-PSMA-1007 for Imaging

In addition to the development of PSMA used therapeutically, numerous PSMA tracers dedicated to tumor imaging are being developed [89]. Among them, ^18^F-PSMA-1007 (Pylarify^®^) was developed and introduced into clinical practice following its approval by the FDA [90,91]. The rationale to use ^18^F instead of ^68^Ga is based on the limited short half-life of ^68^Ga, the cost of a ^68^Ga generator, and the high positron energy that theoretically could limit the spatial resolution and therefore the diagnostic accuracy in small lesions [90,91,92]. Comparative studies of ^18^F-PSMA-1007 and ^68^Ga-PSMA-11 carried out in mCRPC patients demonstrated a similar potential for detecting prostatic lesions [93,94,95]. ^18^F-PSMA-1007 was also evaluated in pre-clinical GBM models, as PSMA imaging has recently gained attention in this indication. ^18^F-PSMA-1007 imaging revealed a high tumor-to-background ratio in orthotopic mouse models of GBM [96]. This promising observation indicates the potential utility of PSMA imaging in GBM for noninvasive evaluation and monitoring.

**Table 2:** Most recent pre-clinical/clinical development using PSMA-targeting agents.

Radiotracer	Type of Molecule	Disease Model	Study	Reference
^225^Ac-PSMA-617	Peptide	mCRPC mCRPC mCRPC mCRPC	Clinical study (51 patients) Clinical study (26 patients) Clinical study (73 patients) Clinical study (3 cohorts; 91, 40, 18 patients) -Tandem with ^177^Lu-PSMA-617	[66] [67] [68] [69]
^177^Lu-L1	Peptide	PC3 PIP (PC xenografts)	Pre-clinical	[72]
^225^Ac-L1	Peptide	PC3 PIP (PC xenografts)	Pre-clinical	[73]
^213^Bi-L1	Peptide	PC3 PIP (PC xenografts)	Pre-clinical	[73]
^211^At-3-Lu	Peptide	PC3 PIP (PC xenografts)	Pre-clinical	[74]
^177^Lu-EB-PSMA-617	Peptide with EB moiety	mCRPC mCRPC HepG2 xenografts, HCC Adenoid cystic carcinoma	Clinical study (4 patients) Clinical study (28 patients) Pre-clinical Clinical (30 patients)	[77] [80] [57] [81]
^177^Lu-LNC1003	Peptide with EB moiety	22Rv (PC xenografts)	Pre-clinical	[82]
^225^Ac-PSMA-TO-1	Peptide + albumin binder (naphthyl group)	C4-2 (PC xenografts)	Pre-clinical	[83]
^225^Ac-PSMA-DA1	Peptide + albumin binder (iodophenylbutyric acid derivative)	LNCaP (PC xenografts) LNCaP (prostate xenografts)	Pre-clinical Pre-clinical	[84] [85]
^227^Th-BAY 2315497	Antibody	PC cell lines and PDXs VCap, ST1273 (PC xenografts)	Pre-clinical Pre-clinical	[87] [88]

### 2.2. Molecules Expected to Be Translated Soon into Clinical Practice

#### 2.2.1. Fibroblast Activation Protein Inhibitor (FAPI)

Unlike many clinically available tracers that directly target tumor cells, the imaging of the fibroblast activation protein (FAP) allows for the detection of cancer-associated fibroblasts (CAFs). FAP is highly expressed by CAFs, and 90% of epithelial tumors and metastases are positive; meanwhile, it is absent in normal adult tissues [97,98,99]. The presence of CAFs and FAP expression have been reported to be strongly associated with metastasis and poor prognosis in various tumor types, making FAP a suitable target for tumor imaging and therapy [100,101]. Recently, several clinical trials were performed using FAP inhibitors (FAPIs) or FAP-targeted antibodies labeled with ^68^Ga or ^18^F for PET imaging and have been recently described in detail by Huang et al. [102]. Clinical evidence suggests that FAPIs may be a molecular imaging tool that complements and is superior to ^18^F-FDG in specific indications, such as primary or metastatic PDAC and liver tumors [103,104,105,106]. Our review focuses exclusively on novel theranostic radiotracers targeting FAP. The current pre-clinical development regarding FAPI focuses on the evaluation of β- or α-emitters (Table 3).

##### Development of β- and α-Emitter Labeled FAPIs

Thomas Lindner et al. were the first to explore the therapeutic potential of FAPIs by radiolabeling them with ^177^Lu (^177^Lu-FAPIs). Of these, FAPI-04, a quinoline-based small molecule, was selected for further development, due to its high tumor accumulation and low activity in healthy tissues of tumor-bearing mice [107]. In this study, a first-in-human study was performed in a patient with metastatic breast cancer after therapy with ^90^Y-FAPI-04. One injection resulted in significant pain reduction. Subsequent studies in mouse models of PDAC showed significant growth delay after a single injection of ^225^Ac-FAPI-04, further supporting its potential as a therapeutic agent [108]. FAPI-04 (^68^Ga-FAPI) was also used to detect early lesions in an orthotopic model of PDAC, using PDXs tumors [109]. This molecule was also labeled with ^131^I and ^211^At for PRRT in gliomas. An intra-tumoral injection of ^131^I-FAPI-04 or ^211^At-FAPI-04 significantly delayed the growth of xenograft tumors [110,111]. A dosimetry study using ^177^Lu-FAPI-04 was conducted on metastatic solid tumors and showed the safety of a low-dose injection [112]. Nonetheless, a short retention time in the tumor was reported, highlighting the necessity of developing other radiotracers to further enhance treatment efficacy.

Among them, FAPI-46 proved to be a more suitable theranostic agent due to its higher retention time compared to FAPI-04 [113]. ^177^Lu-FAPI-46 and ^225^Ac-FAPI-46 were studied in mouse models of PDAC and showed dose-dependent antitumor efficacy [114]. In clinical settings, ^177^Lu-FAPI-46 was shown to be safe (compassionate use) in 18 patients with advanced solid tumors [115]. More recent publications reported the use of ^90^Y-FAPI-46. Of note, 47 cycles of ^90^Y-FAPI-46 were administrated to 21 patients with various solid tumors. Eight out of twenty-one patients had a PET response, encouraging its further development [116]. A phase I/IIa study aiming to evaluate the safety, tolerability, dosimetry, and preliminary activity of ^177^Lu-FAPI-46 is ongoing (LuMIERE trial, NCT04939610).

In pre-clinical studies, FAPI-46 was compared to another one, FAP-2286. The results showed that ^177^Lu-FAP-2286 led to higher tumor retention and showed better tumor control than ^177^Lu-FAP-46 [117].

Other FAPI molecules include the quaric-acid-based FAP inhibitors DOTA.SA.FAPi and DOTA.(SA.FAPi)_2_, which were recently evaluated in the clinic for PET imaging (^68^Ga-DOTA.SA.FAPi). ^68^Ga-DOTA.SA.FAPi has been found to be effective in identifying more metastatic lesions than ^18^F-FDG in patients with metastatic breast cancer [118]. A comparison between ^177^Lu-DOTA.SA.FAPi and ^177^Lu-DOTA.(SA.FAPi)_2_ revealed that the latter had a higher tumor-absorbed dose and led to longer survival in patients with solid tumors [119]. ^177^Lu-DOTA.(SA.FAPi)_2_ was also evaluated in radioiodine-refractory differentiated thyroid cancer (15 patients) [120]. Four patients had a partial response, and three had stable disease after 45 cycles of ^177^Lu-DOTA.(SA.FAPi)_2_. This modality opens new avenues for the treatment of aggressive radioiodine-refractory aggressive cancer. Clinical trials for other indications are pending to assess the potential of this radiotracer for the treatment of solid tumors.

##### Modification of FAPIs to Enhance Tumor Targeting

Recent work in the field of FAPIs molecules also includes the modification of radiotracers, aiming at enhancing tumor retention to overcome limitation of previously cited molecules. One strategy involves the creation of a bivalent FAP ligand, such as ND-bisFAPI [121]. Micro-PET imaging conducted on mice bearing GBM demonstrated a higher tumor retention with ^177^Lu-ND-bisFAPI than ^177^Lu-FAPI-04. Preliminary therapeutic experiments showed a more favorable antitumor response with ^177^Lu-ND-bisFAPI than with ^177^Lu-FAPI-04.

In parallel to such a strategy, biochemical optimizations in FAPIs tracers were performed by adding albumin-binding components. The addition of Evans Blue dye to the FAPI complex (^177^Lu-EB-FAPI-B1) showed prolonged blood uptake and high tumor retention in mice models of GBM, which led to tumor-growth inhibition [122]. Two clinical phase II trials are currently ongoing in patients with advanced and refractory solid tumors (NCT05410821 and NCT05400967). The same pre-clinical results were obtained for tumor growth when using other albumin-binding FAPIs (^177^Lu-TEFAPI06/07), which could be promising tracers, even if no clinical studies have yet been conducted [123]. This strategy was also investigated by Meng et al., using novel albumin-FAP ligands (FSDD0I, FSDD1I, and FSDD3I) in PDX models of HCC [124]. The higher tumor retention of ^68^Ga-FSDD0I compared to the historical ^68^Ga-FAPI-04 led to the subsequent development of ^177^Lu-FSDD0I. This molecule remains to be fully evaluated in pre-clinical models.

In conclusion, despite their high potential, these molecules need further investigation in clinical trials to fully evaluate their potential to improve the management of patients.

**Table 3:** Recent development of FAP-targeting theranostics agents.

Radiotracer	Type of Molecule	Disease Model	Study	Reference
^90^Y-FAPI-04	Quinoline-based	HT-1080 xenografts (fibrosarcoma) Patient: metastatic breast cancer	Pre-clinical and first-in-human studies	[107]
^177^Lu-FAPI-04	Quinoline-based	Metastatic advanced stage cancer	Clinical (4 patients)	[112]
^131^I-FAPI-04	Quinoline-based	U87MG xenografts(glioblastoma)	Pre-clinical	[110]
^211^At-FAPI-04	Quinoline-based	U87MG xenografts(glioblastoma)	Pre-clinical	[111]
^225^Ac-FAPI04	Quinoline-based	PANC-1, MIA PaCa-2 xenografts (PDAC)	Pre-clinical	[108]
^177^Lu-FAPI-46	Quinoline-based	PANC-1 xenografts (PDAC) Relapsed or refractory cancers (10 patients)	Pre-clinical Clinical	[114] [115]
^90^Y-FAPI-46	Quinoline-based	Solid tumors (110 patients)	Clinical	[116]
^225^Ac-FAPI-46	Quinoline-based	PANC-1 xenografts (PDAC)	Pre-clinical	[114]
^177^Lu-FAP-2286	Quinoline-based	HEK293 and PDX (sarcoma)	Pre-clinical	[117]
^177^Lu-DOTA.(SA.FAPI)2	Bivalent FAP	Different tumors (7 patients) Thyroid cancer (15 radioiodine-refractory patients)	First in humans First in humans	[119] [120]
^177^Lu-ND-bisFAPI	Bivalent FAP	A549 xenografts (lung cancer)	Pre-clinical	[121]
^177^Lu-EB-FAPI-B1	Quinoline-based and modified with EB	U87MG xenografts(glioblastoma)	Pre-clinical	[122]
^177^Lu-TEFAPI06/07	Quinoline-based and modified with albumin-binder	HT-1080 xenografts (fibrosarcoma)	Pre-clinical	[123]
^177^Lu-FSDD0I	Quinoline-based and modified with IPBA	PDXs (HCC)	Pre-clinical	[124]

#### 2.2.2. Gastrin-Releasing Peptide Receptor (GRPR)

Recent evidence has shown that the gastrin-releasing peptide receptor (GRPR), also known as bombesin receptor subtype 2 (BB2), holds great promise as a target for theranostic applications. GRPR is primarily expressed in pancreas [125]. When it binds to its ligand (Gastrin-releasing peptide, GRP), GRPR activation lead to multiple physiological processes including the release of gastro-intestinal hormone [126]. GRPR expression has been identified in prostate, breast or gastric cancers [127]. This observation, along with the low physiological expression of GRPR, provides a compelling rationale for employing GRP-derived fragments in theranostics.

The historical development of GRPR-targeting radiotracers initially employed agonists as carriers. However, agonists demonstrated low tumor targeting efficiency and less favorable pharmacokinetic profiles, which restricted their practical application [128,129]. To overcome these challenges and enhance the efficacy of GRPR-targeted radiotracers, recent research endeavors have shifted their focus towards antagonist molecules. The two most promising radiopharmaceuticals, ^68^Ga-RM2 and ^68^Ga-NeoB, have been successfully evaluated for the ability to bind GRPR-expressing tumors with high sensitivity [130,131,132,133,134]. 

Recently, the therapeutic counterparts of these radiotracers have undergone clinical evaluation. ^177^Lu-RM2 was assessed in patients with mCRPC who were ineligible for ^177^Lu-PSMA PRRT [129]. ^177^Lu-RM2 was found to be suitable for therapy, characterized by a high tumor uptake and rapid clearance from healthy organs. However, it was observed that the strong antitumor efficacy might be limited by the metabolic instability of RM2. In a study involving five healthy patients, only 19% of intact tracer was detected one hour post-injection [135]. To address this issue, a substitution of L-tryptophan with α-methyl-L-tryptophan was performed, leading to an improvement in the stability of the radiopharmaceutical (^177^Lu-AMTG) and resulting in a higher tumor-to-background ratio. Nonetheless, clinical studies need to be conducted to determine its superiority.

Significant advancements have been made using ^177^Lu-NeoB radiotracer. Pre-clinical studies conducted on mouse models of PC demonstrated a high tumor uptake and favorable pharmacokinetics [136]. In mouse models of gastrointestinal tumors, ^177^Lu-NeoB exhibited high antitumor efficacy, leading to complete tumor regression in some cases, while sparing healthy tissues [137]. Repeated administration of ^177^Lu-NeoB was well-tolerated in mice that support the subsequent evaluation of the compound in the clinic [138]. The ongoing Neoray clinical trial aims to investigate the safety, tolerability, pharmacokinetics, and antitumor activity of ^177^Lu-NeoB in patients with solid tumors that expressed GRPR based on ^68^Ga-Neob PET imaging [139]. The eagerly awaited clinical results will shed more light on the potential of this treatment.

## 3. Major Recent Advances in the Theranostics Field

**Figure 1:**
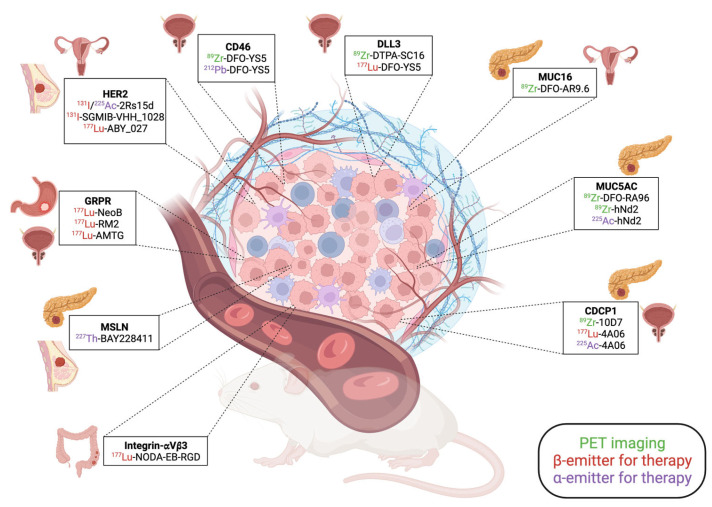
Summary of the recent pre-clinical development in the theranostic field.

Alongside the molecules that have already improved patient care or hold promise for the near future, the development of novel pharmaceuticals and the exploration of innovative strategies, including combination approaches with immunotherapies, are now proposed. The recent molecules developed for theranostic application are presented in Figure 1.

### 3.1. New Theranostic Agents for Solid Tumors

#### 3.1.1. Human Epidermal Growth Factor Receptor 2 (HER2) Targeting

The oncogenic potential of HER2 has been well established in breast cancer [140]. The HER2 antibody trastuzumab has been part of the therapeutic armamentarium of clinicians since its approval in 1998 and remains a standard of therapy for HER2+ breast cancer patients [141]. Beyond breast cancer, HER2 expression has been observed in other solid tumors, such as gastric, lung, colorectal, or brain tumors [142,143]. The measurement of HER2 expression using PET-imaging-dedicated radiotracers has been widely evaluated in clinical practice, using antibodies, nanobodies, or affibodies [22,144,145].

Recent research in this field has primarily focused on optimizing nanobodies for targeted radionuclide therapy. Nanobodies have been reported to offer high specificity and improved tumor tissue penetration compared to traditional antibodies [146]. The 2Rs15d nanobody was recently radiolabeled with the β-emitter ^131^I and with the α-emitter ^225^Ac and evaluated for the treatment of brain metastatic lesions originating from ovarian and breast carcinomas [147]. A weekly injection of ^131^I-2Rs15d or ^225^Ac-2Rs15d improved the survival of mice bearing trastuzumab-resistant tumors. The therapeutic efficacy of ^225^Ac-2Rs15d was also found to be superior to trastuzumab in a intraperitoneal ovarian cancer model [148]. Another nanobody was recently evaluated and compared to 2Rs15d. The tumor uptake of ^131^I-SGMIB-VHH_1028 was reported to be higher than ^131^I-SGMIB-2Rs15d in xenografts of ovarian and breast carcinoma, resulting in significant tumor-growth delay [149]. One of the main problems with the use of nanobodies as a therapeutic agent is their high renal retention. In a later study, the multiple administration of [^131^I]SGMIB-VHH_1028 resulted in urolithiasis. This nanobody was also labeled with ^211^At (^211^At-VHH_1028). With a single dose of 3.0 MBq, ^211^At-VHH_1028 led to complete tumor regression in three of four mice bearing BT474 breast cancer cell xenografts [150]. The same antitumor effect was also observed when ^131^I-NM-02, another nanobody, was used in HER2-positive xenografts [151]. ^177^Lu-ABY-027 affibody was also evaluated for its ability to treat HER2-positive tumors and showed higher efficacy when combined with trastuzumab in comparison to monotherapies [152].

Clinical studies on the antitumor efficacy and safety of antibody-derived radiotracers will determine their therapeutic potential.

#### 3.1.2. Recent Developments for Prostate Cancer 

##### CD46 as an Alternative to PSMA?

The field of PC remains an active field of research since PSMA expression can be heterogeneous and ^177^Lu-PSMA is not curative, despite the prolonged PFS and OS of patients. Recent evidence has highlighted the high plasticity of PC following therapeutic pressure caused by anti-androgen therapy or radiotherapy. Epithelial cells can transdifferentiate into a neuroendocrine phenotype, rendering the tumor ineligible for PSMA PRRT [153,154]. Approximately 17% of mCRPC cases were found to undergo this transdifferentiation, which is strongly associated with shortened survival [155]. CD46 emerges as a relevant target, as it can be expressed in both epithelial and neuroendocrine PC [156]. CD46 is a membrane-bound complement regulatory protein that protects the cells from complement-cytotoxic death and plays a key role in metastatic disease [157,158]. YS5, an antibody directed against CD46, has shown high therapeutic potential in addressing both adenocarcinoma and neuroendocrine PC [156]. A phase I clinical study is ongoing using this carrier as a microtubule inhibitor-based antibody drug conjugate (NCT03575819). This antibody, ^89^Zr-DFO-YS5, was then used for PET imaging of CD46 in xenograft models of PC. Strong and specific uptake was observed in both adenocarcinoma and neuro-endocrine PCs. This makes it a potential candidate as an imaging agent and companion biomarker in PCs [159]. Its therapeutic potential was also investigated by using ^212^Pb-DFO-YS5 in several models of PCs, including PDXs. A single dose of ^212^Pb-DFO-YS5 (0.37 MBq) significantly inhibited tumor growth and prolonged the survival of mice, opening the path for clinical translation [160].

##### DLL3 as an Emerging Target in PCs

Alongside CD46, Delta-like canonical Notch ligand 3 (DLL3) is another promising target for neuro-endocrine PCs. DLL3 is found to be expressed in approximately 80% of neuro-endocrine PCs and lung carcinomas, making it an attractive candidate for therapeutic interventions [161,162]. Various therapeutic approaches targeting DLL3 are currently under evaluation [163]. PRRTs targeting DLL3 have also been recently evaluated in pre-clinical studies. A humanized antibody, ^89^Zr-DFO-SC16, was successfully validated as an imaging tracer for DLL3 in lung adenocarcinoma xenografts [164]. This imaging tracer has shown specific uptake in neuro-endocrine-derived tumors, outperforming other imaging tracers, such as ^68^Ga-PSMA-11 or ^68^Ga-DOTA-TATE, which failed to accurately image these tumors [165]. The therapeutic ^177^Lu-DTPA-SC16 was evaluated in neuro-endocrine PC tumors in a dose-escalated study. A single dose of ^177^Lu-DTPA-SC16 (27.75 MBq) led to complete tumor response, making it a potent candidate for PC [166]. The encouraging results observed were further corroborated by Tully et al. in lung cancers that express DLL3 [167]. Nevertheless, additional research, including toxicology studies, is imperative to fully validate the potential of these radiopharmaceuticals.

#### 3.1.3. Recent Developments for PDAC 

Recent research efforts have also been made regarding theranostics development in PDAC. Notably, mucin proteins such as Mucin-16 (MUC16) and Mucin-5AC (MUC5AC), as well as membrane protein CUB (for complement C1r/C1s, Uegf, Bmp1) domain-containing protein-1 (CDCP1), have garnered significant therapeutic interest during the past 3 years. 

##### MUC16

MUC16 is one of the most well-known mucins, primarily because of its CA125 epitope, which serves as a biomarker for serous ovarian cancer [168]. Historically, most of the anti-MUC16 antibodies developed for targeted therapy purposes harbored a fluorophore or a cytotoxic drug [169,170]. A new class of antibodies has emerged which targets different epitopes, leading to signaling disruption [171,172]. Among these, AR9.6 stands out for its ability to reduce MUC16-induced oncogenic signaling. ^89^Zr-DFO-huAR9.6 was recently evaluated in murine models of MUC16-expressing ovarian cancer and PDAC [173]. This tracer showed a remarkable uptake in primary and metastatic lesions from PDAC, with tumor uptake reaching approximately 70% of injected dose/g 6 days post-injection. Such a molecule could represent a valuable companion tool for MUC16-directed therapy.

##### MUC5AC

Genomic analyses identified MUC5AC as one of the most differentially expressed mucin genes in abnormal pancreatic tissue in comparison to normal tissue [174]. In a study conducted by Henry et al., they used an antibody (RA96) directed against MUC5AC and demonstrated an increase in its expression from pancreatic intraepithelial neoplasia to PDAC [175]. Uptake of the PET tracer ^89^Zr-DFO-RA96 demonstrated a high uptake and tumor-to-background ratio 6 days post-injection in PDAC tumors. This suggests that ^89^Zr-DFO-RA96 could be a promising candidate for the noninvasive detection of PDAC. Other theranostic antibodies, ^89^Zr-labeled hNd2 (NMK89) and ^225^Ac-labeled hNd2 (NMT25), were recently developed and showed high tumor accumulation, while tumor growth was delayed [176]. Additional research is required to assess their sensitivity, specificity, and usefulness for the management of PDAC.

##### CDCP1

CDCP1 has emerged as a crucial player in tumor progression and is strongly correlated with a poor prognosis in PDAC [177]. Pre-clinical studies focus on its targeting for theranostics. The ^89^Zr-10D7 antibody allows for the detection of small lesions of ovarian cancer and PDAC in xenograft models [178,179]. Another antibody, 4A06, was recently developed and labeled with ^89^Zr for imaging or with ^177^Lu- and ^225^Ac for targeted therapy purposes. Encouragingly, ^177^Lu-4A06 and ^225^Ac-4A06 showed a pronounced antitumor effect in a single-dose study [180]. Recent studies reported the high therapeutic efficacy of ^177^Lu-4A06 in pre-clinical models of bladder cancer and mCRPC that exhibit a high expression of CDCP1 [181,182]. As for other antibody-derived radiotracers, expanded studies are warranted.

### 3.2. Development of Bispecific Molecules

Another promising area of research is the recent development of bispecific molecules engineered to simultaneously target two different tumor-associated antigens. Indeed, the application of monomeric receptors, such as PSMA or FAP radiotracers, is limited due to tumor heterogeneity or suboptimal in vivo pharmacokinetics. Alongside the modifications discussed in Section 2, the development of bispecific molecules could be a valuable tool to efficiently image and treat tumors. 

#### 3.2.1. PSMA-FAPI

The dual imaging of PSMA and FAP was recently evaluated by several authors, using different agents. So far, all the developed radiotracers are dedicated to PET imaging. Wang et al. recently developed a bispecific molecule based on the PSMA-617 and FAPI-04 structure, ^68^Ga-FAPI-PSMA [183]. This novel radiotracer showed high potential for the noninvasive imaging of FAPI+ and/or PSMA+ tumors at 1 h post-injection in pre-clinical mouse models. Other ^68^Ga-radiolabeled bispecific molecules were evaluated, but their high blood uptake limited their ability to image tumors [184]. ^18^F-labeled bispecific molecules were also investigated for PET imaging. Specifically, ^18^F-PSMA-FAPI-01 and ^18^F -PSMA-FAPI-02 showed increased tumor uptake in comparison to the monomeric radiotracers ^18^F-FAPI-42 and ^18^F-PSMA-BCH, with an optimal pharmacokinetics profile [185]. Finally, ^64^Cu-FP-L1, another PET radiotracer, displayed prolonged tumor uptake in models expressing PSMA, FAP, or both [186]. Their full potential needs to be evaluated in clinical practice, and the development of a theranostic pair is awaited.

#### 3.2.2. PSMA-GRPR

Novel bispecific-targeting PSMA and GRPR were developed and evaluated by Lundmark et al., following labeling with ^111^In and ^68^Ga for SPECT and PET imaging, respectively [187,188]. ^111^In-BQ7812 and ^68^Ga-BQ7812, bispecific molecules consisting of a urea-based PSMA inhibitor combined with the GRPR-antagonist RM26 were successfully evaluated to image tumor-bearing mice (PSMA- and GRPR-positive). So far, no theranostics application has been reported.

#### 3.2.3. Other Developments of Dual-Targeting Molecules

Other recent developments in the field of bispecific molecules have centered on the integrin αvβ3 ligand RGD (arginin–glycin–aspartic acid). Integrins are heterodimeric transmembrane complexes consisting of two subunits (α and β), which are able to form at least 24 different complexes. Among these, αvβ3 was the first to be found abundantly expressed in several solid tumor types and proposed as a target for both imaging and therapy [23,189,190,191]. In the field of nuclear medicine, many radiotracers have been derived from the Arg-Gly-Asp (RGD) sequence, a well-known ligand of integrin αvβ3 [23]. Recent developments regarding integrin αvβ3 are presented in Section 3 of this review. RGD-derived radiotracers have also been subjected to modification to generate bispecific molecules. One such molecule, ^68^Ga-FAPI-RGD, was recently evaluated and favorably compared to ^68^Ga-FAPI-02 and ^68^Ga-RGDfK for its ability to target PDAC tumors in pre-clinical models [192]. Building up on solid pre-clinical data, a first study in humans was conducted in six patients with solid tumors. Rapid, high, and prolonged tumor uptake, associated with high tumor-to-background ratios, was found with ^68^Ga-FAPI-RGD, making it a promising tool for tumor diagnosis. A clinical study carried out on 22 patients and recently published by Zhao et al. demonstrated an improved lesion detection rate and metastatic delineation of ^68^Ga-FAPI-RGD in comparison to ^18^F-FDG [193]. Similar observations were reported in a pilot exploratory study on patients (*n* = 51) with suspected lung cancers [194]. With the stated advantages, the FAPI-RGD should be further explored for therapeutic applications.

### 3.3. Combination of PRRT with Immunotherapies

The potential of PRRT to be combined with other therapies, particularly with immunotherapies, can constitute one of the most significant achievements in modern oncology. Combination therapies have been a cornerstone of cancer treatment for a long time, leading to superior therapeutic effects and aiming to limit the emergence of drug resistance [195]. The powerful tools that are radiotherapy and immunotherapy can be synergistically combined to achieve prolonged responses in cancer treatment. Several reports demonstrated that radiotherapy could have immunomodulatory effects by releasing tumor antigens that can trigger an immune response, upregulating immune-stimulatory molecules and promoting the infiltration of immune cells [25]. However, careful management of the timing, sequencing, and dosing of both approaches is necessary to achieve the best therapeutic results while minimizing adverse effects. In this way, several clinical trials are ongoing [196]. Along with radiotherapy, PRRT can also play a crucial role in combination therapies.

#### 3.3.1. Combination with FDA-Approved Radiotracers 

##### DOTA-TATE and Immunotherapies

Aicher et al. recently introduced a novel therapeutic concept using short-interval low-dose ^177^Lu-DOTA-TATE in combination with ICIs (pembrolizumab, an anti-PD-L1 antibody) (SILD-PRRT protocol). This protocol, which involves up to six doses of 3.5 GBq per cycle at 3–6 weeks intervals, was applied in combination with pembrolizumab to restore response to ICIs in patients with Merkel cell carcinoma who were previously refractory to ICIs [197]. While other trials are needed to validate this concept, this observation opens a new therapeutic window for ICIs-refractory patients. A recent study showed the importance of the sequential use of this combination therapy in neuroendocrine tumor models xenografted in humanized mice [198]. Higher antitumor responses were observed in PRRT-pretreated mice, followed by anti-PD1 (Nivolumab) antibodies. This result was consistent with high ^68^Ga-NOTA-hGZP tumor uptake, indicating the presence of CD8+ effector cells. Moreover, a phase I clinical trial conducted in nine patients with lung cancer demonstrated the safety and signs of antitumor activity [199]. These studies pave the way for the subsequent use of such therapies that need to be investigated in patients.

##### PSMA and Immunotherapies

Alongside these combination therapies, clinical trials are ongoing, aiming to evaluate the safety, tolerability, and efficacy of ^177^Lu-PSMA-617 in combination with pembrolizumab in patients with mCRPC (NCT03658447, NCT03805594, and NCT05150236). Preliminary results from the NCT03805594 study were communicated, showing that a priming dose of ^177^Lu-PSMA-617, followed by pembrolizumab, was tolerated and led to a prolonged response in a subset of patients without mutational burden [200]. The scientific community is eagerly awaiting the results of the phase II clinical trial. This concept has also been explored in relation to targeted α-therapy. ^225^Ac-PSMA-617 and PD-1 blockade synergistically improve the therapeutic outcome of mice bearing syngeneic PC, laying the groundwork for such a combination [201].

#### 3.3.2. Other Promising Combinations Recently Evaluated in Pre-Clinical Studies

Other combination regimens using PRRT and ICIs were evaluated for their ability to impair tumor growth in pre-clinical settings. Recent studies are presented here.

##### Mesothelin (MSLN) and Combination Therapy

Mesothelin was reported to be expressed by more than 50% of solid tumors, including PDAC and ovarian or breast cancers [202,203]. Various approaches to target mesothelin are currently under clinical investigation [204]. Nuclear medicine has also taken the path of proposing anti-mesothelin PRRT as a potential treatment option. A monoclonal antibody, ^227^Th-BAY 2287411, was recently evaluated in vitro and in vivo in cell lines from ovarian cancer and PDAC, as well as on a PDX model of ovarian and breast cancer [205]. ^227^Th-BAY 2287411 demonstrated significant antitumor efficacy in a single-dose or multiple-dose regimen. Its immunostimulatory properties were also evaluated in combination with anti-PD-L1 therapy in immunocompetent mice bearing tumors [206]. ^227^Th-BAY 2287411 treatment was found to activate innate and adaptative immune systems, priming the tumor for combination treatment with ICIs. These findings could drive the development of combination strategies in clinical practice.

##### Integrin αvβ3

A similar approach has also been reported with the use of radiolabeled RGD. One of the most promising RGD peptides dedicated to PRRT, ^177^Lu-NODA-EB-RGD, an RGD combined with Evans Blue dye, was evaluated in PDX models of lung cancers. Administering a single injection of ^177^Lu-NODA-EB-RGD resulted in the complete eradication of tumor growth [207]. A therapeutic regimen based on ^177^Lu-NODA-EB-RGD and anti-PD-L1 was proposed in a murine colon cancer model. The sequential order of this combination was found to be critical, as a concurrent blockade of PD-L1 and PRRT led to tumor-growth control and increased overall survival (OS) and provided protection against tumor rechallenge [208]. Nevertheless, additional studies are needed to fully characterize the potential of such a strategy.

##### NM600

NM600, an alkylphosphocholine analog with desirable tumor selectivity, was also evaluated in combined strategies. ^177^Lu-NM600 alone was proved to be a suitable theranostic agent for breast cancer, with optimal pharmacokinetics [209]. Additionally, ^90^Y-NM600 treatment activates the stimulator of interferon genes (STING) and induces immune susceptibility markers, giving the rationale for its combination with ICIs [210]. ^90^Y-NM600 was then evaluated for enhancement of tumor response to ICIs (anti-CTLA4) in models of cold tumors. After combined treatment, half of treated mice exhibited a complete tumor response compared to none of the mice in the group that received PRRT or ICIs alone [211]. This study highlighted the potential of PRRT to make cold tumors suitable for ICIs and opens new avenues for therapeutic combinations.

##### Other Combinations with ICIs

Several other combinations are currently under development, such as the targeting of melanin with several radiotracers labeled with ^177^Lu, ^131^I, ^213^Bi, or ^225^Ac and combined with ICIs [212,213,214]. All of them show remarkable tumor-growth delay in immunocompetent mouse models of melanoma and deserve clinical evaluations. Alongside these tracers, ^177^Lu-DOTA-folate also showed impressive antitumor effects when combined with anti-CTL4 antibodies [215]. All of these combinations have high potential to eradicate tumor lesions but need to be fully validated in clinical trials.

## 4. Conclusions

As research and innovation propel the field forward, theranostics is set to play an increasingly vital role in revolutionizing cancer treatment and patient care. The recent successes of Luthatera^®^ and Pluvicto^®^ have opened up exciting possibilities for developing new theranostics agents. Repurposing these agents for other indications and enhancing their tumor-targeting abilities are areas of keen interest that could significantly enhance patient care in the near future. To further bolster this therapeutic arsenal, ongoing clinical trials with radiolabeled-FAP and Neob hold promise. Additionally, the recent pre-clinical advancements in new radiotracers offer potential solutions for relapsing diseases or highly devastating conditions such as PDAC. Furthermore, α-therapy shows significant potential in the treatment of various solid tumors. Nonetheless, there are still unanswered challenges to address. One such challenge is the control of the offspring generated by α-emitters such as ^225^Ac, ^227^Th, or ^211^At, which could potentially restrict the practical use of this therapy. It is crucial to strike a balance between the therapy’s cytotoxic effects and its tolerability, which remains to be determined.

Additionally, one of the most significant accomplishments in nuclear medicine in recent years is its potential combination with ICIs. By harnessing the complementary mechanisms of radiotherapy and immunotherapy, researchers and clinicians are paving the way for innovative treatment strategies that hold great promise in improving cancer outcomes. As these clinical trials continue, they will help define the true potential of theranostics and its role in the clinician’s armamentarium.

## Abbreviations

CDC1P	CUB domain containing protein 1
CRPC	castration-resistant prostate cancer
CTLA-4	cytotoxic T-lymphocyte antigen 4
FAPI	fibroblast activation protein inhibitor
FDA	Food and Drug Administration
GBM	glioblastoma multiforme
GRPR	gastrin-releasing peptide receptor
HER2	Human Epidermal Growth Factor Receptor 2
ICI	immune checkpoint inhibitor
LET	linear energy transfer
LNCaP	Lymph Node Carcinoma of the Prostate
MSLN	mesothelin
MUC16	mucin-16
MUC5AC	mucin-5AC
NET	neuro-endocrine tumor
OS	overall survival
PC	prostate cancer
PDAC	pancreatic ductal adenocarcinoma
PDX	patient-derived xenograft
PD-1	programmed cell death 1
PD-L1	programmed cell death ligand 1
PET	positron emission tomography
PFS	progression-free survival
PRRT	peptide receptor radionuclide therapy
PSMA	prostate-specific membrane antigen
RT	radiotherapy
SARTATE	sarcophagine octreotate
SSTR2	somatostatin receptor 2
TKI	tyrosine kinase inhibitor
